# Low-dose biliatresone treatment of pregnant mice causes subclinical biliary disease in their offspring: Evidence for a spectrum of neonatal injury

**DOI:** 10.1371/journal.pone.0301824

**Published:** 2024-04-05

**Authors:** Kapish Gupta, Jimmy P. Xu, Tamir Diamond, Iris E. M. de Jong, Andrew Glass, Jessica Llewellyn, Neil D. Theise, Orith Waisbourd-Zinman, Jeffrey D. Winkler, Edward M. Behrens, Clementina Mesaros, Rebecca G. Wells

**Affiliations:** 1 Department of Medicine, Division of Gastroenterology and Hepatology, Perelman School of Medicine, University of Pennsylvania, Philadelphia, Pennsylvania, United States of America; 2 Center for Engineering MechanoBiology, University of Pennsylvania, Philadelphia, Pennsylvania, United States of America; 3 Center of Excellence in Environmental Toxicology, Perelman School of Medicine, University of Pennsylvania, Philadelphia, Pennsylvania, United States of America; 4 Division of Gastroenterology, Hepatology and Nutrition, Children’s Hospital of Philadelphia, Philadelphia, Pennsylvania, United States of America; 5 Department of Chemistry, University of Pennsylvania, Philadelphia, Pennsylvania, United States of America; 6 Department of Pathology, School of Medicine, New York University, New York, New York, United States of America; 7 Institute for Gastroenterology, Nutrition and Liver Diseases, Schneider Children’s Medical Center of Israel, Petach Tikva, Israel; 8 Sackler Faculty of Medicine, Tel-Aviv University, Tel-Aviv, Israel; 9 Division of Rheumatology, The Children’s Hospital of Philadelphia, Philadelphia, Pennsylvania, United States of America; The Hormel Institute (University of Minnesota), UNITED STATES

## Abstract

Biliary atresia is a neonatal disease characterized by damage, inflammation, and fibrosis of the liver and bile ducts and by abnormal bile metabolism. It likely results from a prenatal environmental exposure that spares the mother and affects the fetus. Our aim was to develop a model of fetal injury by exposing pregnant mice to low-dose biliatresone, a plant toxin implicated in biliary atresia in livestock, and then to determine whether there was a hepatobiliary phenotype in their pups. Pregnant mice were treated orally with 15 mg/kg/d biliatresone for 2 days. Histology of the liver and bile ducts, serum bile acids, and liver immune cells of pups from treated mothers were analyzed at P5 and P21. Pups had no evidence of histological liver or bile duct injury or fibrosis at either timepoint. In addition, growth was normal. However, serum levels of glycocholic acid were elevated at P5, suggesting altered bile metabolism, and the serum bile acid profile became increasingly abnormal through P21, with enhanced glycine conjugation of bile acids. There was also immune cell activation observed in the liver at P21. These results suggest that prenatal exposure to low doses of an environmental toxin can cause subclinical disease including liver inflammation and aberrant bile metabolism even in the absence of histological changes. This finding suggests a wide potential spectrum of disease after fetal biliary injury.

## Introduction

Biliary atresia (BA) is a fibrosing cholangiopathy presenting in the neonatal period. The incidence of BA ranges from 1 in 3,500 [1] to 1 in 18,000 [2, 3] live births worldwide, with a higher prevalence in certain populations, particularly in East-Asia [4, 5], and Latin America [6]. Despite advances in the medical and surgical management of newly-diagnosed newborns, a significant proportion of patients progress to liver failure and require liver transplantation.

Although extrahepatic bile duct (EHBD) obstruction and fibrosis, bile duct inflammation, and ultimately liver fibrosis and cirrhosis are the most commonly-appreciated manifestation of BA, serum bile acids are also significantly elevated [7–9]. However, it is not known whether bile metabolism is altered independently of EHBD obstruction and the resulting abnormal enterohepatic circulation.

The exact cause of BA is unclear and the disease is likely multifactorial, with both genetic and environmental factors playing a role [10]. Several studies suggest that prenatal or perinatal viral infections, exposure to toxins, or abnormalities in the immune system contribute to the development of this disorder [6]. We have shown that biliatresone, a toxin implicated in the development of BA in Australian livestock, leads to EHBD damage in larval zebrafish and mouse EHBD explants, providing a proof-of-concept for a toxic etiology [11–13]. Two groups reported that mice treated with biliatresone intraperitoneally at 80 mg/kg on postnatal day 1 (P1) develop a BA-like phenotype, including jaundice and bile duct obstruction [14, 15], although prenatal administration to pregnant mothers resulted in either no obvious phenotype (~40–50 mg/kg orally) or prenatal lethality (~30–50 mg/kg given once intraperitoneally) in their pups [14].

In order to define the different manifestations of toxic prenatal hepatobiliary injury, we treated pregnant mice with a low concentration of biliatresone (15 mg/kg orally for each of two days) then evaluated liver and bile duct histology, liver and serum bile acid profiles, and the liver immune profile of the pups. We report that there is a disconnect between histological and biochemical abnormalities, suggestive of a broader spectrum of neonatal biliary disease than previously appreciated.

## Results

### Pups from biliatresone-treated mothers are asymptomatic and histologically normal

**Fig 1:**
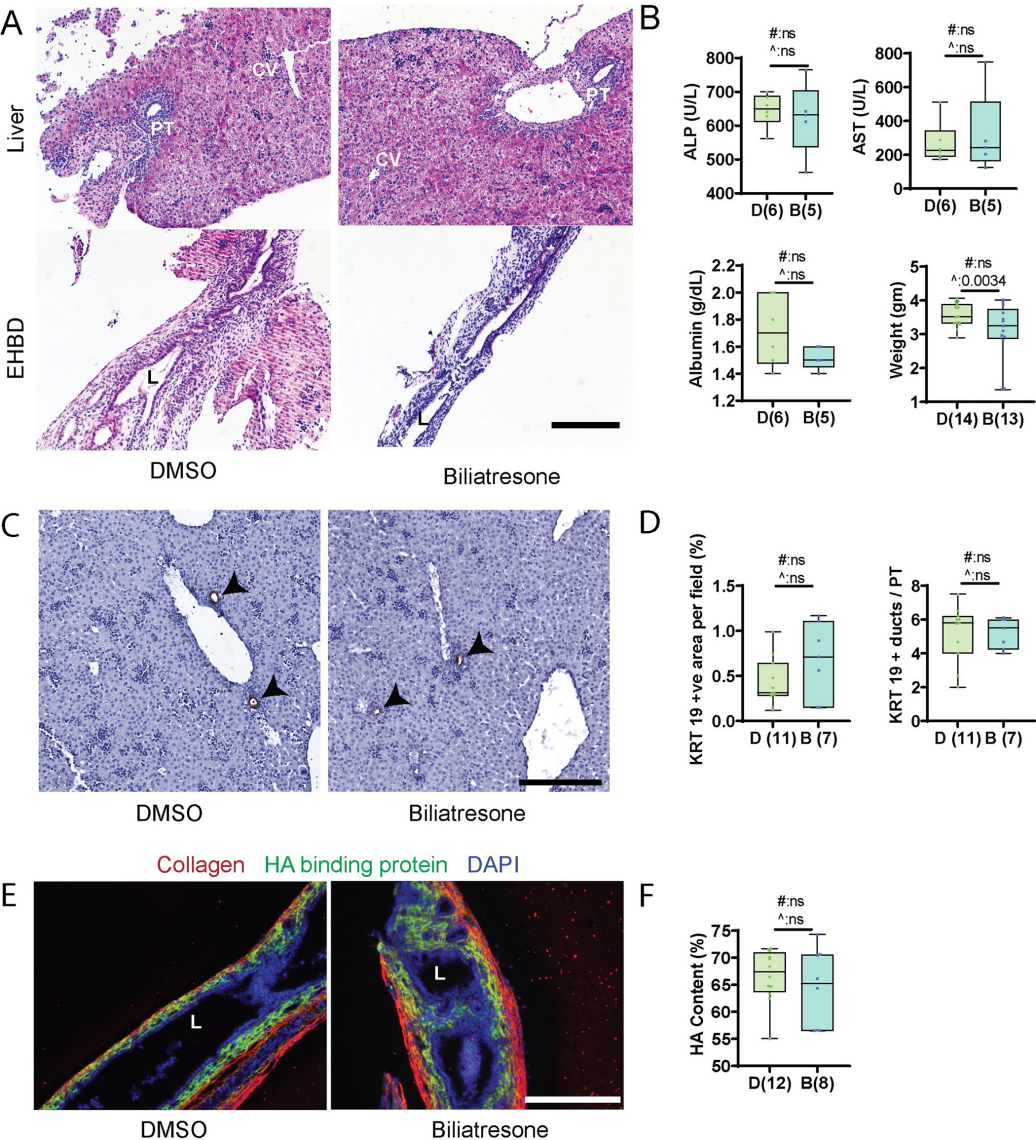
Prenatal biliatresone exposure does not result in significant histological or biochemical abnormalities in P5 pups. (A) Representative H & E staining of liver and EHBD sections isolated from P5 pups born to control and biliatresone-treated mothers (n = 8). (B) Serum biochemistry and physical parameters for P5 pups. The number of pups is shown in parentheses; given the small amount of serum available, a random subset of serum samples was assayed. (C) Liver sections were also stained for KRT 19. The black arrowheads mark bile ducts. (D) Quantification of the number of KRT 19-positive foci per portal triad and KRT 19-positive area per field, with the number of pups indicated in parentheses. (E) Representative images showing staining for HA-binding protein (green), collagen (red) and DAPI (blue) in P5 pups born to control and biliatresone-treated mothers. (F) Quantification of submucosal area stained by HA-binding protein. The number of pups is shown in parentheses. D: DMSO, B: Biliatresone, PT: portal triad, CV: central vein, L: lumen. All scale bars, 200 μm. #: t-test p value, ^: F test p value, ns: not significant.

**Fig 2:**
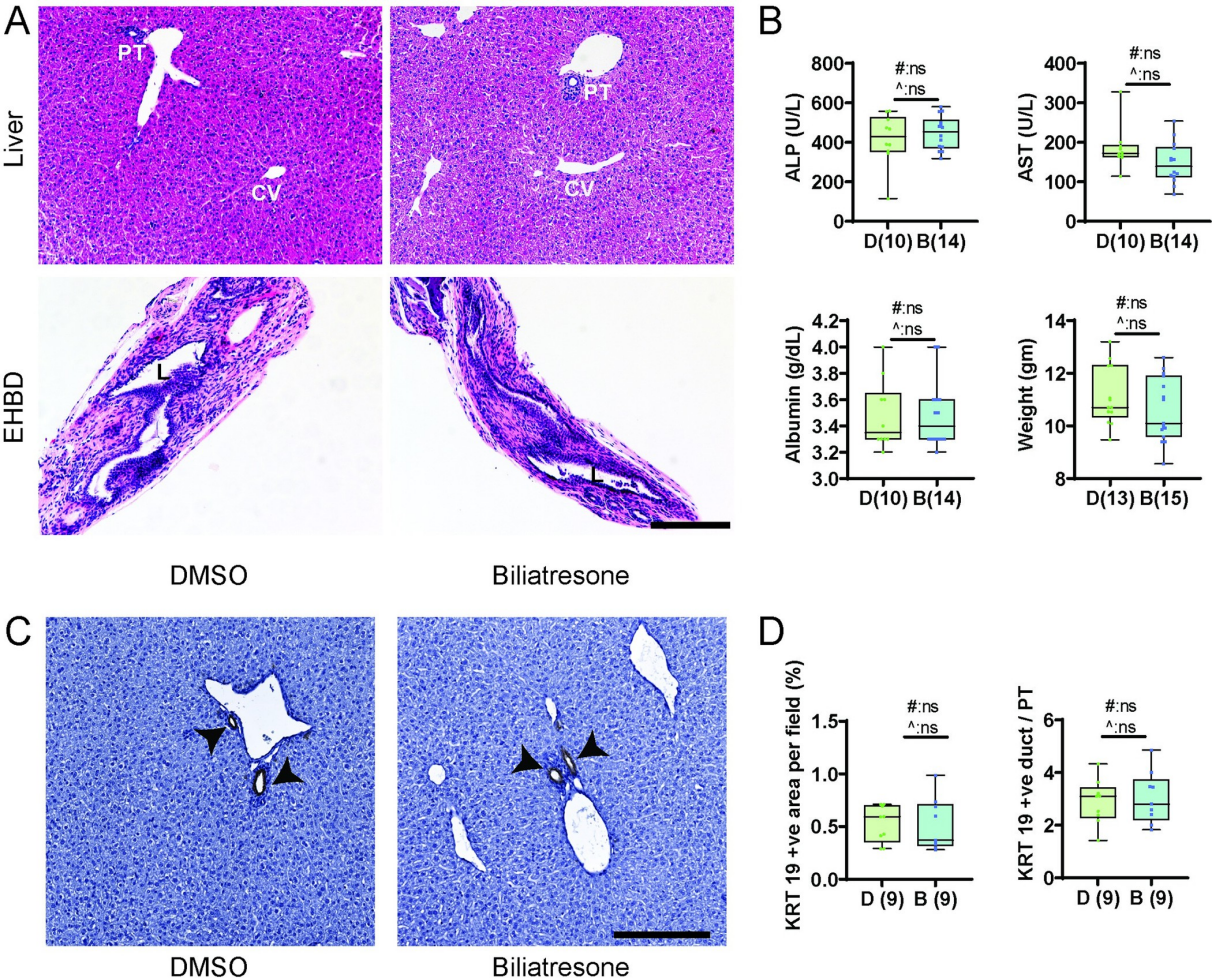
Prenatal biliatresone exposure does not result in significant histological or biochemical abnormalities in pups at P21. (A) Representative H & E staining of liver and EHBD sections isolated from P21 pups born to control and biliatresone-treated mothers (n = 8). (B) Serum biochemistry and physical parameters of P21 pups. The number of pups is shown in parentheses. C) Liver sections were also stained for KRT 19. The black arrowheads mark bile ducts. (D) Quantification of the number of KRT 19-positive foci per portal triad and KRT 19-positive area per field, with the number of pups indicated in parentheses. D: DMSO, B: Biliatresone, PT: portal triad, CV: central vein, L: lumen. All scale bars, 200 μm. #: t-test p value, ^: F test p value, ns: not significant.

Pups born to mothers treated with either low-dose biliatresone or vehicle all appeared healthy at P5 and P21. None of the pups developed jaundice, displayed distress or died, although the treated group at P5 showed higher variability in weight (Fig 1B). There were no histological abnormalities in either liver or bile duct sections observed for either group (Figs 1A and 2A) as determined through the grading method outlined in the Methods section entitled "Histology Assessment”. No ductular reaction was observed in either group (Figs 1C, 1D, 2C and 2D). Stains for hyaluronic acid (HA) and type I collagen, major components of the extracellular matrix that increase in the setting of fetal/neonatal injury and are potential indicators of fibrosis, showed no difference between pups of control and biliatresone-treated mothers, consistent with a lack of damage to the EHBD (Fig 1E and 1F) [16]. Liver biochemistries were assessed in both groups of pups at P5 and P21 and on average, the two groups of pups showed no significant differences (Figs 1B and 2B).

### Bile acid profile shows differences following biliatresone treatment

**Fig 3:**
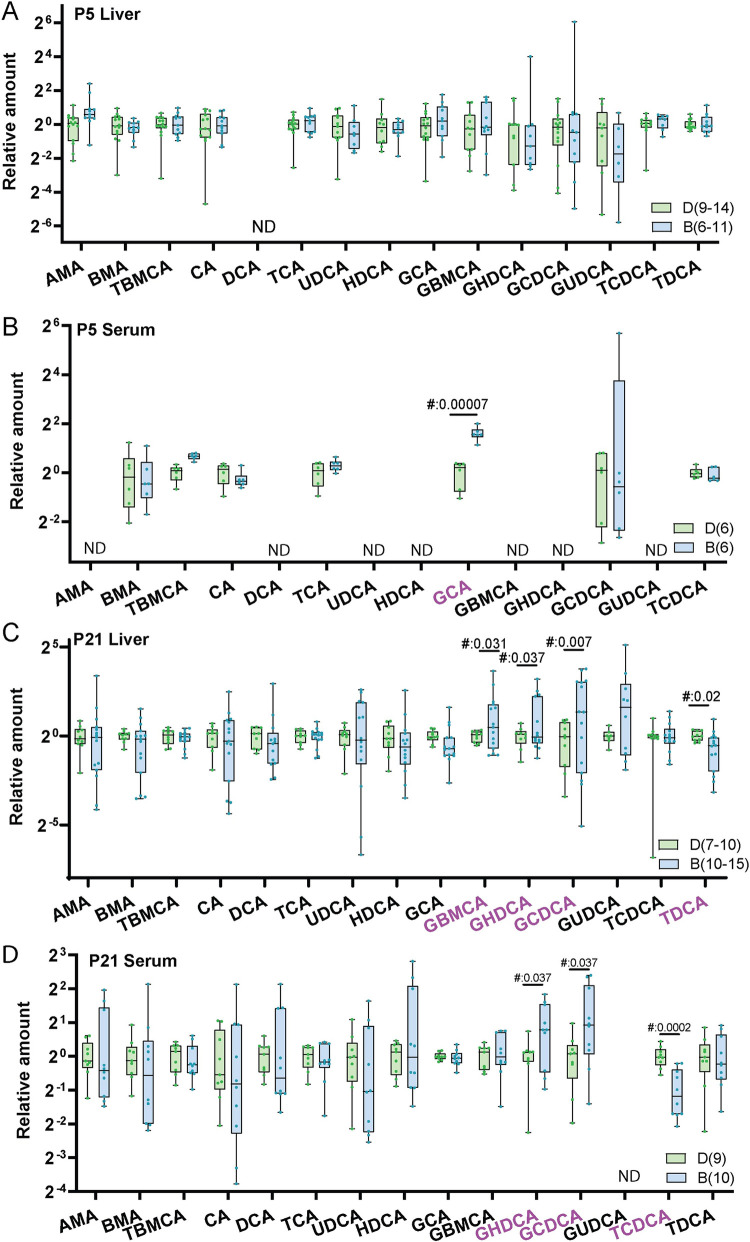
Glycine-modified bile acids were increased in pups from biliatresone-treated mothers. (A-D) Relative amounts of various bile acids from liver and serum from P5 and P21 pups. Individual bile acids were normalized to the corresponding mean from control pups. The graphs display only those bile acids that were at detected in at least 5 samples, while the remaining bile acids are denoted as not determined (ND). The names of bile acids that are significantly different between control and biliatresone-exposed pups are highlighted in purple.

We measured liver and serum bile acid levels in pups from both control and biliatresone-treated pups to determine whether prenatal biliatresone treatment had any impact on bile acid metabolism. At P5, we found no significant differences in the liver bile acid profile between the two groups of pups (Fig 3A). In contrast, we did observe a change in the serum bile acid profile, with elevated levels of glycocholic acid (GCA) in the treated group compared to the control group (Fig 3B). This suggests that low-dose toxin exposure in the prenatal period may alter bile acid metabolism.

At P21, we found that glycine-conjugated bile acids were increased in both liver and serum of the pups of the biliatresone-treated group compared to the control group. In liver, glycine-β-muricholic acid (GBMCA) (2.7±0.8 fold), glycohyodeoxycholic acid (GHDCA) (2.4±0.68 fold), and glycochenodeoxycholic acid (GCDCA) (4.4±1.2 fold) were significantly increased (Fig 3C), while in serum, GHDCA (1.8±0.3 fold) and GCDCA (2.4±0.5 fold) were significantly increased (Fig 3D). There were also significant decreases in taurodeoxycholic acid (TDCA) (0.71±0.12 fold) in liver and taurochenodeoxycholic acid (TCDCA) (0.5±0.07 fold) in serum. However, taurine-conjugated bile acids still comprised more than 80% of the total bile acid pool. The alterations in the bile acid profile were consistent across litters.These findings suggest that low-dose biliatresone treatment in the prenatal period may lead specifically to an increase in glycine-conjugated bile acids and that these abnormalities continue to worsen well past the time of exposure.

### Differences in liver immune profile following biliatresone treatment

**Fig 4:**
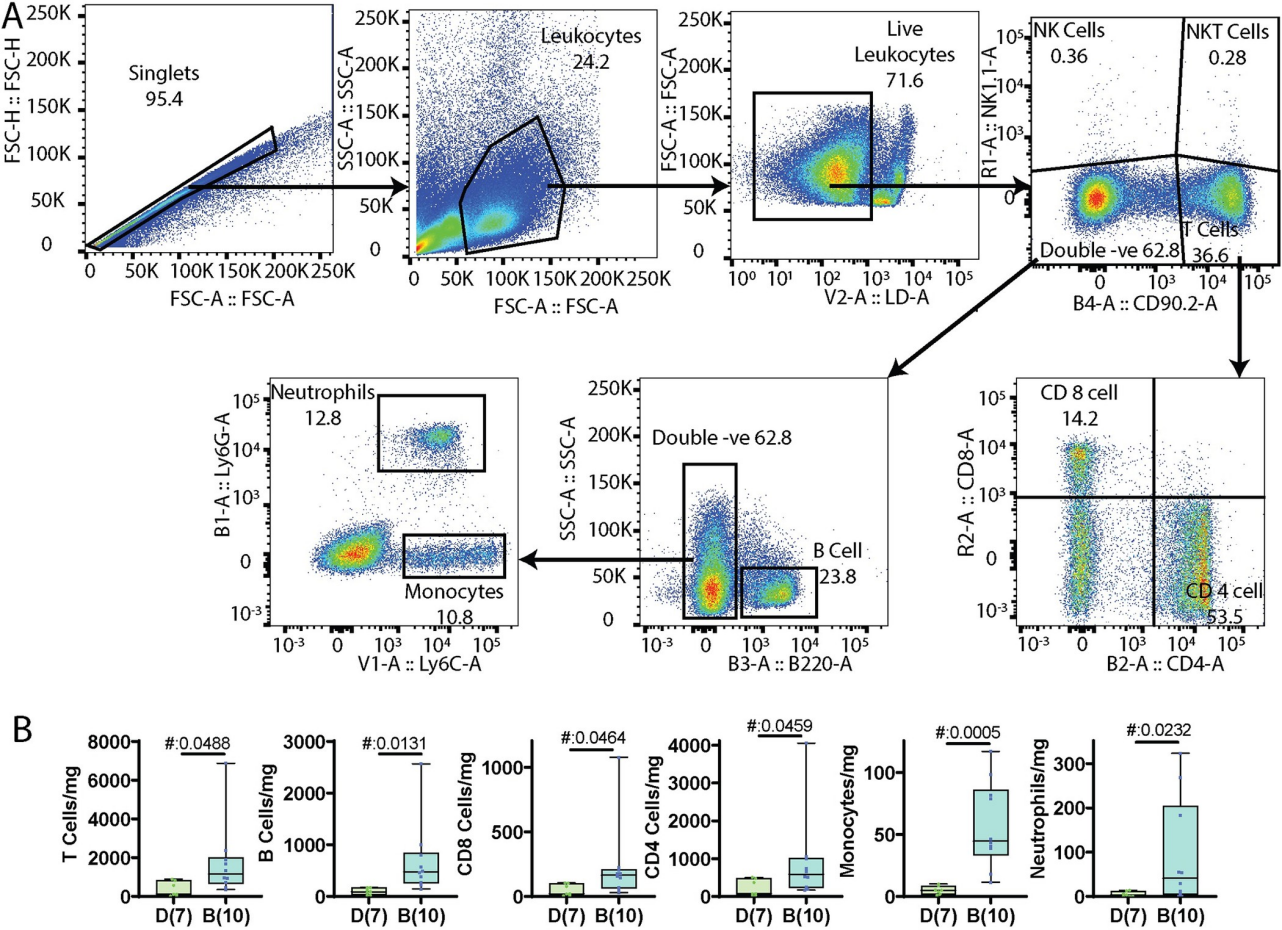
Immune infiltrates were increased in pups from biliatresone-treated mothers. (A) Panel used to identify B cells (Live, NK1.1-, CD90.2-, B220+), T-cell populations (Live, NK1.1-, CD90.2+, CD4+ or CD8+), neutrophils (Live, NK1.1-, CD90.2-, B220-, Ly6G+Ly6C+) and monocytes (Live, NK1.1-, CD90.2-, B220-, Ly6G-, Ly6C+). (B) Quantification showing numbers of T-cells, CD4 cells, CD8 cells, B-cells, monocytes and neutrophils in livers isolated from P21 pups. The number of pups is shown in parentheses. D: DMSO, B: biliatresone, #: t-test p value.

Bile acids have been shown to regulate inflammation [17], and thus we examined livers from the P21 mouse pups for the presence of inflammatory cells (Fig 4A). We found that all P21 animals from biliatresone-treated mothers had significantly elevated liver immune cells compared to those from control mothers (Fig 4B). Of the cells identified (B and T cells, neutrophils, and monocytes), B cells and monocytes showed the most significant increase. One pup had unusually high B and T cells, and another pup from a different litter had a high monocyte count. The data remain statistically significant even with the exclusion of these outliers.

B cells have been implicated in bile duct injury in the rhesus rotavirus (RRV) model of BA [18], and the finding that they were significantly increased in the livers of pups from biliatresone-treated mothers suggests that even low-dose biliatresone treatment may be associated with an increased risk of bile duct injury. Monocytes, which are similarly involved in inflammation and immune responses, were also significantly increased in the livers of the treated pups.

### Biliatresone treated mothers were similar to control mothers

**Fig 5:**
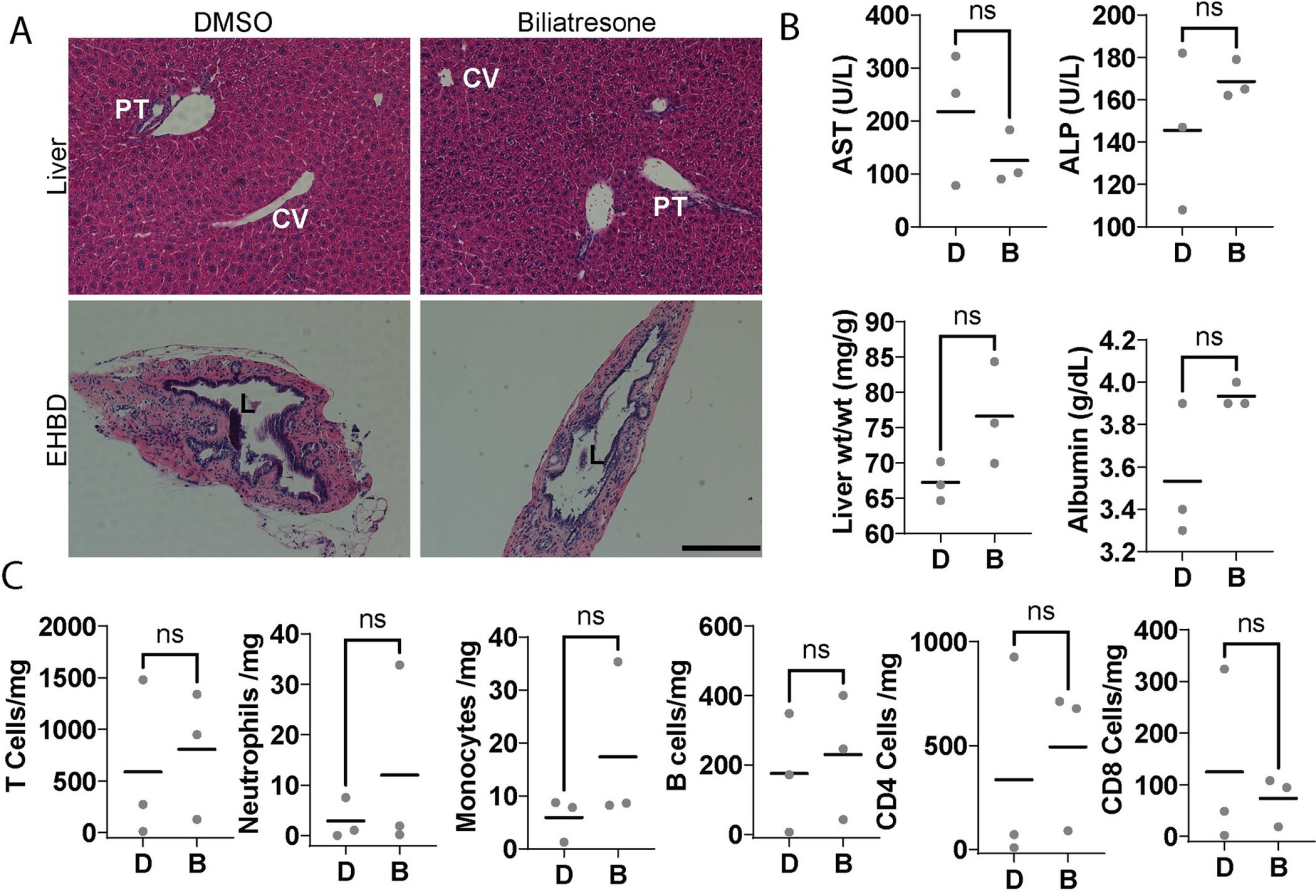
Mothers exposed to biliatresone show no significant histological or biochemical abnormalities or immune infiltration. (A) Representative H & E staining of liver and EHBD sections isolated from control and biliatresone-treated mothers. (B) Serum biochemistries and physical parameters from control and biliatresone-treated mothers. (C) Quantification showing numbers of immune cells in livers isolated from control and biliatresone-treated mothers. N = 3. D: DMSO, B: biliatresone, PT: portal triad, CV: central vein, L: lumen. Scale bar, 200 μm. ns: not significant.

The mothers of BA patients are typically healthy, raising the question of whether low-dose biliatresone treatment has any impact on the mother mice. To investigate this question, we euthanized the mothers when their pups reached P21 and evaluated serum biochemistries, liver histology, and liver immune profiles. We found that mothers treated with either low-dose biliatresone or vehicle appeared similarly healthy, with no obvious phenotypic differences. There were no histological abnormalities observed in liver or bile duct sections from either group, and on average, the two groups had no significant differences in liver biochemistries (Fig 5A and 5B). Furthermore, we found no differences between the groups regarding the presence of immune cells (Fig 5C).

## Discussion

We demonstrate here that exposing pregnant mice to the biliary toxin biliatresone results in persistent elevations in glycine-conjugated serum bile acids in their pups, without histological evidence of EHBD or liver damage. This suggests that biliatresone can alter bile homeostasis independent of EHBD injury.

Developing a physiologically-relevant BA mouse model based on a prenatal insult has been challenging. Although this is likely in part because mice are less prone to develop cholestatic diseases than humans [19], the rarity of BA and the presence of abnormal direct/conjugated bilirubin at birth in many apparently healthy babies who do not develop BA suggests an additional possibility [20]: that the environmental insult responsible for BA causes transient, mild disease in most exposed fetuses, and persistent and progressive disease in only a minority. If this is the case in humans and also holds true in mouse models of BA, finding a BA-afflicted mouse pup even after exposure of the mother to a physiologically-relevant concentration of a toxin or virus might be infrequent [21]. Unfortunately from the perspective of this hypothesis, babies with elevated direct/conjugated bilirubin at birth have not undergone full serum bile acid analyses. Our aim here was to determine–as a means of understanding the full spectrum of disease in toxic fetal biliary injury–whether exposing mothers to low doses of biliatresone would generate a mild, sub-clinical phenotype.Pups born to control and low-dose biliatresone-treated mothers showed no signs of disease, confirming earlier reports [14], and careful histological assessment of EHBDs showed no evidence of damage or recovery post-damage and histological assessment of liver showed no ductular reaction [16]. However, there were several clear differences between the two groups of pups. In the P5 group of pups from biliatresone-treated but not control mothers, two of 13 pups had very low weight compared to the average, resulting in a significantly high variance in the biliatresone-treated population. This suggests that some animals could be more sensitive than others to biliatresone, as was observed for the postnatal biliatresone exposure models developed by Yang et al. and Schmidt et al., which resulted in only a fraction of pups treated with high biliatresone (70–80 mg/kg) developing persistent EHBD damage, with most pups showing either no pathological phenotype at all or jaundice followed by recovery [14, 15].

The abnormal bile acid metabolites that were found in the serum of pups from biliatresone-treated mouse mothers were similar to those reported in the serum of babies with BA. While BA patients undergoing operative cholangiography have elevations in multiple plasma bile acids [22], these abnormalities, which have been observed during the advanced stages of BA, have been attributed to obstruction resulting from bile duct damage. However, Zhou et al. [23] studied dried blood samples obtained 4 days after birth (before the development of overt signs of bile duct damage) from infants who later developed BA, and found that bile acids (mainly GCA) were already elevated. This suggests the possibility that abnormal bile acids contribute to or at least precede clinically-detectable bile duct damage. GCA is the major bile acid present in the human neonatal period and it decreases with age; however, in the study from Zhou et al., GCA remained elevated in BA patients [24, 25]. Our mouse studies identified significant GCA elevations in P5 pups born to mothers administered biliatresone. GCA levels normalized by P21 in mice, but at this age there was a significant increase in other glycine-conjugated bile acids, including GHDCA and GCDCA, in both liver and serum. Glycine modifications are more common in humans than in mice (which favor taurine conjugation), and GCDCA, which results from the modification of chenodeoxycholic acid in humans, is the most abundant bile acid in adults [25]. Increased GCDCA has been linked to cholestasis and cirrhosis [26]. Since bile acids conjugated to taurine are less toxic than those conjugated to glycine, the low levels of glycine-conjugated bile acids in mice may be linked to the low incidence of cholestasis in mice compared to humans. Consistent with this, humanizing mouse bile acids by administering GCDCA leads to collagen deposition and fibrosis in the inducible hepatocellular cholestasis in cholate-fed Atp8b1^G308V/G308V^ mouse model [19], and the presence of GCDCA in biliatresone-treated EHBDs leads to an increase in cholangiocyte injury as well as fibrosis [27]. Thus, the increase in GCDCA observed in mouse pups after maternal treatment with biliatresone is significant as it suggests the potential for increased bile toxicity, which could cause or exacerbate liver and duct damage. TDCA levels in the liver and TCDCA levels in the serum were found to be lower in the pups treated with biliatresone. However, it is unlikely to indicate a large-scale shift from taurine-conjugation to glycine-conjugation of bile acids as tauro-β-muricholic acid (TBMCA) and taurocholic acid (TCA) remained the dominant bile acids, constituting more than 80 percent of the total bile acid pool.

Bile acids are conjugated to glycine or taurine by bile acid-CoA amino acid N-acyl-transferase (BAAT) enzymes; the ratio between the two modifications depends on BAAT selectivity (which varies across species) and by availability of taurine precursors. Murine BAATs are particularly inefficient at conjugating glycine even when taurine availability is reduced [28]. It remains to be seen whether there are any other pathways involved in regulating taurine vs. glycine conjugation. Interestingly, a recent study found that glycine conjugation in mice does not decrease after BAAT knock down, suggesting that alternate pathways are available [29]. These could include bile conjugation by gut microbes and conjugation mediated by the peroxisomal acyltransferases ACNAT1 and ACNAT2 [29]. It is unknown whether BAAT or any of the potential alternate pathways are affected by biliatresone exposure.

Because bile acids are known to regulate inflammation [17], we also investigated the immune profile of P21 pups and observed across-the-board increases in immune cells. The results shown here suggest that biliatresone can activate the immune system, although it is unknown whether it does so independently or through disturbing bile metabolism.

We also assessed whether mouse mothers showed any cholestatic phenotype in response to low-dose biliatresone treatment. Our results suggest that biliatresone has no effect on adult animals, as we found no significant differences in serum biochemistry, liver histology, or immune cell profile between the biliatresone-treated mothers and those treated with the vehicle. These findings are consistent with previous studies, which have shown that adult animals have a relatively robust biliary system that can resist damage [19, 27, 30]. They suggest that low-dose biliatresone treatment does not cause harm to adult mice and that potential effects of the treatment are limited to the offspring.

In conclusion, we show that the pups of mice treated with low-dose biliatresone during pregnancy have altered bile acid and immune profiles similar to those observed in BA patients, even in the absence of significant histological changes. Although biliatresone is unlikely to be a cause of human BA, the data suggest that maternal toxin exposure could lead to a spectrum of fetal hepatobiliary injury, with cryptic changes in bile acid modification pathways reflecting mild damage, and that the number of cases of mild injury could markedly outnumber the number progressing to full BA–potentially an explanation for the rarity of the disease.

## Materials and methods

### Materials

The bile acids ursodeoxycholic acid (UDCA), hyodeoxycholic acid (HDCA),TDCA, TCA, cholic acid (CA), TCDCA, GCA, β-muricholic acid (BMA), TBMCA, α-muricholic acid (AMA), deoxycholic acid (DCA), glycoursodeoxycholic acid (GUDCA), GHDCA, GCDCA and GBMCA as well as an internal standard stock solution containing a mixture of CA-d4, GCA-d4 and DCA-d4 were obtained from Cayman Chemical (Ann Arbor, Michigan, USA). Stock solutions of 1 mg/mL in 100% methanol were diluted to obtain calibration curves for concentrations ranging from 15.6 to 1000 nM. Percoll was obtained from GE Healthcare Life Sciences (Chicago, Illinois, USA). Antibodies against CD4, Ly6G and CD90.2 were obtained from BD Pharmingen (San Diego, CA, USA) and antibodies against CD8α, NK1.1, B220 and Ly6C from BioLegend (San Diego, CA, USA). Goat anti-collagen I antibody for staining was obtained from Southern Biotech (Birmingham, AL, USA). Rabbit anti-Cytokeratin 19 (KRT 19) antibody was obtained from Abcam (Boston, MA, USA). Biotinylated Goat Anti-Rabbit IgG Antibody was obtained from Vector Laboratories, Inc. (Burlington, CA, USA). Biotinylated HA-binding protein for staining HA was obtained from EMD Millipore (Burlington, MA, USA). LIVE/DEAD fixable viability dye was from Life Technologies (Carlsbad, CA, USA). Biliatresone was synthesized as previously described [31]. Stock solutions were made in DMSO and diluted in 1X PBS for gavage.

### Animal experiments

We used BALB/c mice obtained from Jackson Laboratories as animal model. All animal experiments were conducted following the National Institutes of Health policy, and the study was approved by the Institutional Animal Care and Use Committee at the University of Pennsylvania under protocol #804862, ensuring that all procedures were performed ethically and with minimal harm to the animals.

To investigate the effects of biliatresone, pregnant female mice were administered either 15 mg/kg of biliatresone or vehicle (containing an equivalent concentration of DMSO, at 0.3 ml/kg) via gavage on days 14 and 15 post mating. All gavaging procedures were carried out with utmost consideration to ensure humane and ethical treatment throughout the experiment. Animals were closely monitored post-gavaging for any signs of distress, and no instances of stressed animals were recorded during the observation period. We used a dosage of 15 mg/kg of biliatresone in order to avoid pregnancy loss, which was reported by Yang et al. [14] with higher doses. The average litter size in the biliatresone-treated group was 6, comparable to the control group. We chose E14 and 15 for biliatresone treatment in alignment with the time of hepatoblast differentiation and early biliary network formation [32].

Half of the pups from each litter (chosen randomly) were euthanized at P5, and the other half were euthanized at P21. All animals were handled with humane care. Carbon dioxide was used as the primary method of euthanasia according to NIH recommendations for the specific ages. P5 pups also underwent a secondary physical method of euthanasia through decapitation, while other animals underwent cervical dislocation as the secondary method.We did not determine sex given that anogenital distance measurements at P5 lack accuracy, but our random selection of pups for euthanasia at a specific day typically yields a balanced male-female distribution. Mother mice and P21 pups were euthanized on the same day, and blood, EHBDs, and liver samples were collected from all the animals. Placental tissue was not collected. Although in utero exposure is the most likely route of biliatresone toxicity in pups, we could not rule out transfer in milk [33] and therefore kept nursing mothers with pups until P21, then euthanized both.

Serum samples were analyzed for albumin, alkaline phosphatase (ALP) and aspartate transaminase (AST) levels, as well as bile acid concentrations. Liver samples were analyzed for bile acids and immune cells. EHBD and liver samples were fixed and stained for further analysis. There were 13–15 total pups in each group; however, due to limited serum sample volumes, especially in P5 pups, not all analyses could be performed on all samples.

### Histochemistry and immunostaining

After collection, EHBDs and liver samples were fixed in 10% formalin and embedded in paraffin. The embedded samples were then sectioned at 5 μm thickness, and slides were stained for Hematoxylin and Eosin (H&E). Standard protocols were followed for processing the slides.

For antibody staining, EHBD sections were deparaffinized with xylene and rehydrated through a graded series of alcohols and distilled water. Antigen retrieval was performed in 10 mM citric acid buffer (pH 6.0). Sections were blocked with 5% bovine serum albumin and permeabilized with 0.4% Triton X-100 prior to antibody incubation. Sections were stained for collagen I, HA and DAPI as described in [16]. For collagen cy3 anti-goat antibodies and for HA-binding protein, Cy2-streptavidin secondary antibodies were used (1:500, Vector Laboratories).

Liver sections were stained for KRT 19 and labelled with diaminobenzidine. Sections were incubated with 3% H_2_O_2_ to quench endogenous peroxidases and blocked with StartingBlock™ T20/PBS Blocking Buffer (Thermo Fisher Scientific, Waltham, MA, USA) and Avidin D and Biotin Blocking Reagents, prior to incubation with primary KRT 19 antibodies (1:500) overnight at 4°C. The next day, sections were incubated with secondary antibodies (1:500) for 30 minutes at 37°C and visualized using an Avidin-Biotin Complex detection system (Vector Elite Kit, Vector Laboratories, Burlingame, CA, USA). Signals were developed by a diaminobenzidine substrate kit for peroxidases (Vector Laboratories) and counterstained with hematoxylin.

### Histology assessment

For bile duct H&E-stained slides, a qualitative assessment of damage was performed by grading the slides as normal or abnormal based on several features. These features included the presence of luminal debris, marked inflammation, detachment of surface epithelium, and signs of regeneration (including multi-layered surface epithelium and peribiliary gland expansion). Similarly, liver H&E-stained slides were graded as normal or abnormal based on the presence of bile duct damage, ductular reaction and the presence of bile plugs. The grading was performed independently by two researchers, IDJ and NDT, with more than eight animals per group being analyzed.

### Image analysis

Image analysis of stained sections was performed with Fiji ImageJ and QuPath v0.2.0 software. The QuPath selection tool was used to calculate the area of the biliary submucosa that was occupied by HA (based on HA-binding protein staining) relative to the entire submucosal area. As a second measure for the thickness of the HA layer, the width between the lumen and the HA-collagen interface was measured in at least 5 different places, and was adjusted relative to the entire thickness of the bile duct wall.

For KRT 19 stained samples, the QuPath pixel classification tool was utilized to measure the KRT 19 positive area relative to the field area. The number of KRT 19-positive foci per portal triad was counted manually.

### Liver immunology

Intrahepatic leukocytes were isolated by Percoll density gradient centrifugation and stained with LIVE/DEAD fixable viability dye, or with antibodies against CD4, CD8α, NK1.1, B220, Ly6C, Ly6G, and CD90.2. All samples were separated on a MACSQuant flow cytometer (Miltenyi Biotec, Gaithersburg, MD, USA) and analyzed using FlowJo software version 10.6 (Tree Star) (Fig 4A).

### Sample processing for HPLC

Bile acids were extracted from homogenized liver and serum samples as described [34, 35]. Separations were performed on a Waters BEH C18 Column (2.1 mm x 50 mm 1.7 μm). Mobile phase A was water with 0.1% formic acid, and mobile phase B was methanol with 0.1% formic acid at 0.4 mL/min flow. The gradient started at 5% B and was changed to 40% B over 2 min, then to 99% B over 2 min, held constant for 3 minutes then back to the initial composition for equilibration of the column, for a total chromatographic separation time of 12 min. Analysis was conducted on a Thermo Q Exactive HF coupled to an Ultimate 3000 UHPLC interfaced with a heated electrospray ionization (HESI-II) source. The instrument was operated in negative ion mode alternating between full scan from 250–800 *m/z* at a resolution of 120,000 and parallel reaction monitoring at 60,000 resolution with a precursor isolation window of 0.7 m/z. Since sample amounts were limited, not all analyses were performed on all samples. Bile acid values were normalized to average values obtained for control pups in each set of experiments. Some bile acids could not be detected in all samples; for purposes of the analysis, only those bile acids detected in at least 5 samples were considered.

### Statistical analysis

Statistical significance was calculated by one and two-tailed Student’s t-tests. Differences in variance were tested using the F test [36]. The number of samples tested for each experiment is given in parentheses in the graphs. All data are shown as boxplots.
